# Disease Resolution in Chikungunya—What Decides the Outcome?

**DOI:** 10.3389/fimmu.2020.00695

**Published:** 2020-04-28

**Authors:** Priyanshu Srivastava, Ankit Kumar, Abdul Hasan, Divya Mehta, Ramesh Kumar, Chetan Sharma, Sujatha Sunil

**Affiliations:** Vector-Borne Diseases Group, International Centre for Genetic Engineering and Biotechnology (ICGEB), New Delhi, India

**Keywords:** chikungunya virus, incubation phase, acute phase, immunopathology, disease resolution, chronic phase, chikungunya induced arthritis, viral clearance

## Abstract

Chikungunya disease (CHIKD) is a viral infection caused by an alphavirus, chikungunya virus (CHIKV), and triggers large outbreaks leading to epidemics. Despite the low mortality rate, it is a major public health concern owing to high morbidity in affected individuals. The complete spectrum of this disease can be divided into four phases based on its clinical presentation and immunopathology. When a susceptible individual is bitten by an infected mosquito, the bite triggers inflammatory responses attracting neutrophils and initiating a cascade of events, resulting in the entry of the virus into permissive cells. This phase is termed the pre-acute or the intrinsic incubation phase. The virus utilizes the cellular components of the innate immune system to enter into circulation and reach primary sites of infection such as the lymph nodes, spleen, and liver. Also, at this point, antigen-presenting cells (APCs) present the viral antigens to the T cells thereby activating and initiating adaptive immune responses. This phase is marked by the exhibition of clinical symptoms such as fever, rashes, arthralgia, and myalgia and is termed the acute phase of the disease. Viremia reaches its peak during this phase, thereby enhancing the antigen-specific host immune response. Simultaneously, T cell-mediated activation of B cells leads to the formation of CHIKV specific antibodies. Increase in titres of neutralizing IgG/IgM antibodies results in the clearance of virus from the bloodstream and marks the initiation of the post-acute phase. Immune responses mounted during this phase of the infection determine the degree of disease progression or its resolution. Some patients may progress to a chronic arthritic phase of the disease that may last from a few months to several years, owing to a compromised disease resolution. The present review discusses the immunopathology of CHIKD and the factors that dictate disease progression and its resolution.

## Introduction

Chikungunya disease (CHIKD), caused by an arthritogenic alphavirus, chikungunya virus (CHIKV), is becoming a major public health hazard (1, 2). The past few decades have seen the re-emergence of this viral infection as evidenced by epidemics in Africa, Asia, Europe and, in recent years, the Americas (3, 4). The first report of this infection dates back to the 1900s; it was then confused with dengue and was largely underreported (5). The latest outbreaks started during 2005 in the La Reunion islands where *Aedes albopictus* was the primary vector (6–8). It was identified that specific mutations in the viral E1 glycoprotein provided fitness to the virus by reducing its extrinsic incubation period within the mosquito and thereby was transmitted by the vector over a longer period of time (9, 10). Post-2005, the virus spread to different parts of the globe either by travelers or autochthonous outbreaks in tropical and temperate climates involving both mosquito species (9, 11).

CHIKD is primarily a viral infection manifested as a fever with severe arthritic joint involvement. Rightly so, the disease was named “Chikungunya,” which in Makonde means “that which bends up,” emphasizing the excruciating joint pain experienced by the affected individuals which disables movement (12). Even though the infection is classically characterized by fever and joint pains that last for up to a week and sequelae involving joint pains for a few weeks (13), the disease can last up to 2–3 years in a small proportion of patients, affecting the joints and resulting in arthritis-like conditions (14, 15). Recent outbreaks have reported neurological complications in patients stemming from the involvement of the central nervous system (16, 17) as well as an impaired immune system (18, 19). Furthermore, mortality owing to co-morbid conditions was also observed (20, 21). The virus has also been reported to infect through vertical transmission between mother and unborn child and results in complications such as encephalopathy (22, 23).

CHIKD establishes itself and progresses to a prolonged malady involving the joints over a period of time. As the virus infects an individual, there is a period of intrinsic incubation before the clinical symptoms appear and the disease progresses. The disease can then be clinically categorized into acute, post-acute, and chronic phases that can last for a few days up to several months, mainly depending on the individual's immune response to the virus (24, 25). Several studies have established that host immunity can play an important role in disease progression and its resolution after an acute phase of the disease (26–28). The innate immune system has shown to be protective, which may result in early resolution of the disease as evaluated in some reports (29, 30). At the same time, components of adaptive immunity have been reported to be instrumental in mounting the severity of the disease and resulting in the chronic arthritic condition that could last for years (26, 31, 32). Recent research has attributed several viral factors that could also contribute to alleviated chronic conditions, such as persistence of defective viral particles in the host (33) and prolonged infection of synovial macrophages (24).

**Figure 1:**
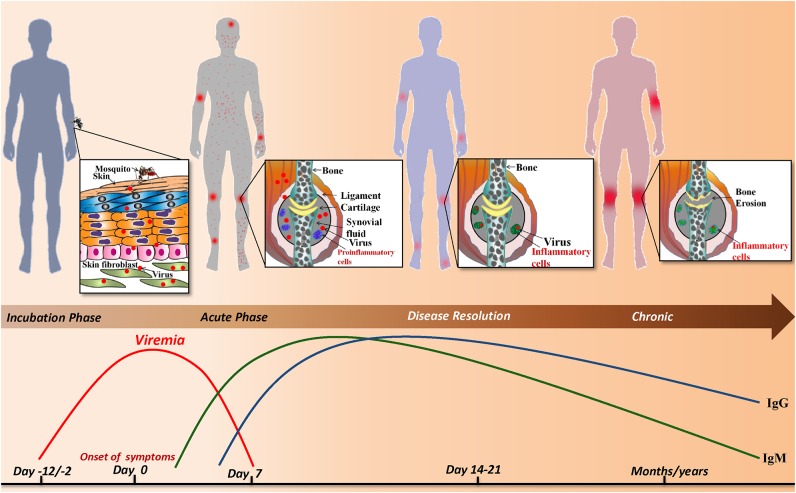
Sequence of events during CHIKV infection: infected *Aedes* mosquito deposits the virus into the dermis and epidermis of the skin. The viral infection is characterized by an incubation period of which is followed by the acute phase during which a rapid rise in viremia occurs and clinical symptoms such as joint pain, fever, maculopapular and petechial rash appear. As the viral load increases the host innate and adaptive immune responses are evoked simultaneously. IgM and IgG levels rise and the virus is resolved from the host. Despite the robust host immune response, viral particles persist in the synovial fluid of joints and alter joint pathology resulting in a chronic phase that lasts for months/years.

The current review is a chronological compilation of the immunopathological events that take place during the acute and post-acute phases of CHIKD that may either lead to resolution of the disease or contribute to an exaggerated immune response, resulting in a full-blown arthritic chronic phase lasting up-to several years (Figure 1).

## Acute Phase

### Early Events Leading to the Establishment of CHIKV Infection

The pre-acute phase or the incubation period of CHIKD ranges from 2 to 12 days (13, 34–38). As an infected mosquito takes a blood meal, it deposits the virus into the epidermis and dermis layers of the skin (39). At the same time, the mosquito also punctures nearby blood vessels, allowing the virus to enter the bloodstream as evidenced by the presence of virus in the sera as early as within 5 min of a mosquito bite (40). At the site of a mosquito bite, the resident cells of the cutaneous immune system defend the host from the pathogen. The cutaneous immune system acts as the first line of defense against the pathogen and is comprised of a heterogeneous mix of cells, such as the lymphocytes, dendritic cells (DCs), and monocytes (39). Gamma delta T lymphocytes (γδ T cells) constitute the most abundant skin-resident T lymphocytes (41) and have demonstrated to play a critical role in cytotoxicity, cytokine secretion, and DC maturation and enhance their function (42, 43). These cells are present in the skin, the local site of infection for CHIKV, and lack the major histocompatibility complex (MHC) restriction; therefore they do not require conventional antigen processing and thus can react to the unprocessed antigens (43). Infection of CHIKV in mice (footpad) led to a significant increase in the prevalence of this T cell subset in foot and popliteal lymph nodes (44). Further, mice deficient in γδ T cells had an increased disease severity accompanied by histopathologic changes (45, 46). Skin resident dendritic cells such as langerhans cells encounter antigens at the site of infection and may disseminate the virus to draining lymph nodes where they interact with the effector T cells, resulting in the initiation of the adaptive immune response (38). Various studies (although contradictory in the explanation of mechanism) have documented the role of bite-induced enhancement of arboviral infection (47–50). Studies have indicated that mosquito saliva has immune-modulatory properties that enhance pathogen establishment within the host by limiting the host immune response (39, 47, 51, 52). One such study of CHIKV infection in mice models observed changes in cutaneous cytokine profiles when inoculating the virus with a needle or mimicking natural infection using mosquitoes. Needle inoculation of the virus into mice resulted in an increased expression of TLR3 and Th1 cytokines (IFN-γ and IL2) whereas infection by mosquito bite elicited a Th2 cytokine response with an increase in IL4 and a reduction observed in TLR3, IFN-γ and IL2 (53). In a different study carried out using dengue virus, similar IFN-γ reduction was observed when the virus was inoculated with mosquito saliva as compared to direct needle inoculation of the virus (54). Similarly, research on the immune modulatory properties of mosquito saliva showed that mice previously sensitized with a salivary gland extract of mosquitoes have more robust cellular infiltration than non-sensitized mice. This study suggests that mosquito saliva has the ability to differentially regulate cellular microenvironment around the bite site (55). Another recent study correlated the initial events of mosquito bite and viral inoculation with subsequent development of systemic disease, highlighting an important role for cutaneous innate immune response. The study observed that stromal cells and macrophages were the main cellular targets for semliki forest virus replication at the initial bite sites, but these cells did not express markers of interferon induction upon infection. Dermal DC's, that are less abundant in the dermis, were major activators of type I IFN response at the initial inoculation site and therefore were major players in limiting viral replication and clinical progression (56).

However, other studies have argued that bite-induced enhancement is too early a process to activate and engage cells of the adaptive immune response (47, 51). Infections with Semliki forest virus aided or unaided by mosquito saliva showed no reduction in IFN-γ levels, suggesting no perturbations of cutaneous antiviral immunity (47). The authors showed that in the presence of saliva, two major events were observed. First, the development of edema that retained the virus at the bite site thus delaying the dissemination of the virus to draining lymph nodes, slowing the initial innate immune response activation and enhancing cutaneous cell replication. Second, an influx of neutrophils attracted by cutaneous cells was observed beginning from 90 min of infection in the presence of mosquito saliva (47). Although neutrophils were themselves refractory to arboviral infection, they attracted cells of the myeloid lineage as new targets for viral replication. Myeloid cells are also responsible for spreading the virus from the initial site of infection, and neutrophil depletion at the bite site reduced viral dissemination (47). The importance of type I IFNs during early infection is evidenced by IFNAR-deficient mice models of CHIKV infection in which mice died within 3 days post CHIKV infection. Skin fibroblast cells upon infection by CHIKV virus were found to be major producers of type I IFNs (Specifically IFN-β). IFN production in these cells was triggered by the activation of extracellular as well as intracellular pathogen sensing receptors, such as TLR3 and MyD88, as a result of infection, thus leading to a strong anti-viral defense early during CHIKV infection (57).

### Establishment of the Acute Phase of Infection

The beginning of the acute phase is marked by viral dissemination from the local site of replication to the primary sites of CHIKV infection in the host body (24). Hallmarks of the acute phase are peak viremia, manifestation of clinical symptoms, and production of neutralizing antibodies (58–60). The acute phase of CHIKD typically lasts for 7–14 days (37, 61, 62). In both humans and murine models, the general observation is that CHIKV-induced immunopathology is considered the primary mediator of damage and persistent pain.

Viral replication in fibroblast cells (primary site of infection) is initially limited by a rapid and robust induction of interferons (IFN-β) and downstream signaling molecules. Interferons help to recruit immune cells that sense the viruses within the infected cells. This sensing may involve the specific pathogen-associated motifs like viral nucleic acids, that are recognized by pattern recognition molecules/receptors (PPRs); these receptors can be cytoplasmic [e.g., Class I—NOD-like Receptors (NLRs), retinoic inducible gene-I like receptors (RLRs), melanoma differentiation factor (MDA)-5, laboratory of genetics and physiology (LGP)-2, cyclic GMP-AMP synthase (cGAS), Gamma-interferon-inducible (IFI) protein-16 and Class II—Protein kinase R (PKR), DNA sensor AIM2 and 2′-5′-oligoadenylate synthase (OAS)-3] (63), or endosomal (e.g., Toll-like receptors; TLRs) (64). It was observed that inflammasome-specific molecules such as NLRP3 of the NLR family were associated with peak inflammatory symptoms during CHIKV infection and these effects could be reversed upon the usage of caspase-1 or NLRP3 inhibitors (65). Previously, the role of inflammasomes in CHIKV infection was explored through *in-vitro* studies using siRNA targeting caspase-1 that showed enhancement in CHIKV replication with silencing (66).

Members of RIG-1-like helicase families such as RIG-1 and MDA5 play a significant role in the activation of IFN-I response (67). These PPRs from Class I detect dsRNA or 5′-triphosphate RNA in the cytosol and induce IFN production. It was shown that stimulation with 5′-triphosphate RNA generates a robust antiviral response against CHIKV through RIG-1 pathway with the help of transcription factor IRF3 (67). Simultaneously, TLR3 gene expression was found to be significantly elevated and associated with up-regulation of several downstream molecules including IRF-1, IRF-3, IRF-7 and OAS-3, IFN-β, and TFN-α production (68). An association of TLR3 with CHIKV replication was shown by an agonist of TRIF-dependent signaling of interferon induction (69) and TRIF knockout mice showed high viral titer with increased foot swelling in comparison to WT infected mice (70). The role of TLR7 was also investigated by MyD88 knockout mice that showed high viremia but no difference in the severity of disease in comparison to WT infected mice (70), implying that MyD88 dependent pathway may not be involved in disease progression. However, another study showed a significant role of MyD88 signaling in controlling viral dissemination (57). Transcriptional analyses of peripheral blood from CHIKV infected patients showed a high expression of viperin in monocyte (71). The role of viperin was further evaluated using Rsad2 knockout mice that showed a significant direct correlation of the expression of this molecule with respect to higher viremia and joint inflammation (71).

Despite the robust and rapid immune response against the virus in permissive cells, CHIKV effectively evades the cellular control mechanisms. In fibroblast cells, for example, CHIKV dodges the interferon response by inducing a translational shut off (27). Additionally, the cytopathic nature of CHIKV induces apoptosis (72). Fibroblast and stromal cells, for example, undergo apoptosis within 24 h post-infection (72, 73). The virus utilizes this cellular response to increase its rate of infection and since the viral particles are sequestered within the apoptotic blebs, it escapes recognition by the immune system. Engulfment of the apoptotic blebs by neighboring phagocytic cells such as macrophages promotes infection in a non-inflammatory or dormant manner (74). As the virus replicates, it simultaneously invades nearby blood vessels. Evidence has demonstrated the infection of blood monocytes by CHIKV virus (75). The virus continues replication, achieving a peak in viremia during the acute phase.

### Immunopathology of Acute Phase

**Figure 2:**
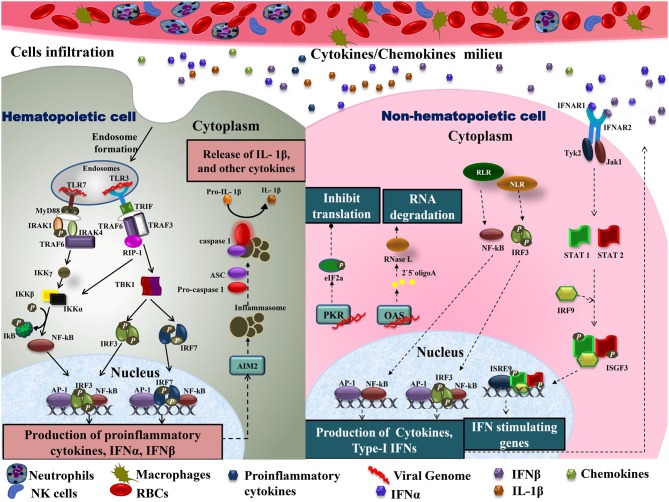
Immune cell infiltration in CHIKV infected tissues: the primary target organs during CHIKV infection include liver, spleen, joints, and kidney. As the virus is disseminated to these sites during acute phase, it infects non-hematopoietic cells. Active viral replication within these cells generate viral dsRNA and ssRNA which is recognized by RLR and NLR leading to downstream activation of NF-κB and phosphorylation of IRF3 results in type-I IFNs and cytokine production within cells. Increase amount of IFNs in extracellular matrix activates IFNARs leading phosphorylation and dimerization of STAT1 and STAT2 in the presence of IRF9 results in activation of IFN stimulating genes. The presence of viral dsRNA and pro-inflammatory cytokines within these cells also activates OAS and PKR. Downstream of PKR activation is the generation of initiation factor eIF2a leading to a translational arrest. On the other hand, OAS once triggered generates RNase L which degrades viral RNA. In case of hematopoietic cells, endosomal TLRs recognize viral dsRNA. Downstream of TLR7, MyD88-IRAK-1-IRAF4-TRAF6 complex is recruited leading to phosphorylation of IkB. Likewise, signaling downstream of TLR3 involves the recruitment of TRIF. TRIF further interacts with TRAF6 and TRAF3. This complex recruits kinase RIP-1 triggering IRF3 and IRF7 phosphorylation downstream of TBK1 activation as well as IkB phosphorylation. These events lead to NF-κB dependent induction of pro-inflammatory cytokines, IFN-α and IFN-β. Cytokines within these cells also produce inflammasomes downstream of AIM2, which promotes apoptosis by cleaving pro-caspase1 to caspase1. Active IL-1β production is also enhanced during this event. The pro-inflammatory cytokines are also released extracellularly, generating a cytokine rich milieu and attracting other immune cells causing cellular infiltration. TLR, Toll-Like receptor; MyD88, myeloid differentiation primary response 88; TRAF, tumor necrosis factor receptor (TNFR)-associated factors; IKB, nuclear factor of kappa light polypeptide gene enhancer in B-cells inhibitor; TRIF, TIR-domain-containing adapter-inducing interferon; IRF, interferon regulatory factor; TBK1, TANK-binding kinase 1; NF-κB, nuclear factor k-light-chain enhancer of activated B cells; IFN, interferons; RLR, RIG-1 like receptor; NLR, NOD-like receptor; OAS, 2′5′ oligoadenylate synthetase; PKR, dsRNA-dependent protein kinase R; eIF2a, eukaryotic initiation factor 2 alpha subunit; RNase L, 2-5A dependent ribonuclease L; AIM2, absent in melanoma; IL-1β, Interleukine 1β; STAT, signal transducer and activator of transcription; IRF9, interferon regulatory factor 9; ISGF, IFN-stimulated gene factor; IFNAR, interferon-alpha/beta receptor.

As CHIKV infection spreads, host immune responses are activated by engaging various subsets of myeloid cells as well as lymphocytes to control this spread. A major event in the acute phase is infiltration of macrophages, neutrophils, natural killer (NK) cells, and other lymphocytes to the primary sites of infection, mainly joints and muscles, leading to hypertrophy in the cells of synovial lining as well as surrounding synovial vessels (76) that manifests as arthralgia in the patients (24, 77). This cellular infiltration is mediated by pro-inflammatory cytokines and chemokines rich milieu at the sites of infection (30) (Figure 2). CCL2 (MCP-1) is a major chemokine that is produced by various cell types like endothelial, fibroblasts, epithelial, smooth muscle, mesangial, astrocytic, monocytic, and microglial cells (78–81). Macrophages, being the major source of CCL2, regulate the infiltration of monocytes and NK cells to the site of infection. Cessation in macrophage recruitment by using Bindarit, a modulator of CCL2 production, has shown reduced inflammation in synovial and skeletal muscle tissues (82). Another study showed contrasting results in which CCR2^−/−^ mice exhibited increased foot swelling and cartilage erosion upon CHIKV infection (83). Macrophages are also known to be the reservoirs of CHIKV in later stages of infection (24), which possibly plays an important role in prolonged inflammation. CCR2 plays an important role in controlling inflammation and musculoskeletal pathology by maintaining the balance of monocytes and neutrophil infiltration to the infected tissues (83). CCR2^−/−^ mice upon CHIKV infection showed enhanced arthritic symptoms without any significant change in viremia, as compared to wild type mice. Also, the monocyte/macrophage infiltrates were replaced by an enhanced neutrophil infiltration, followed by eosinophils infiltrates as well (83). These results suggest that neutrophils are one of the key mediators of arthritic symptoms and tissue damage in CHIKV infection by releasing granules carrying antimicrobial molecules and producing reactive oxygen species (ROS) due to an oxidative burst, which is also a well-established feature of neutrophils in other models of inflammation (84, 85). Neutrophils are also known to produce an anti-viral effect in an alternate way, by forming neutrophils extracellular traps (NETs) through a process called NETosis. *In vitro* CHIKV infection in neutrophils isolated from mice resulted in the formation of NETs through TLR7 and ROS dependent mechanism. Also, the correlation of NETosis with susceptibility and viral load has been shown in IFNAR^−/−^ mice model and CHIKV infected patients, respectively, suggesting the anti-CHIKV role of NETosis (86). NETs are also known to activate plasmacytoid dendritic cells (pDCs), a subtype of dendritic cells, which secrete high levels of IFN-I (87, 88). In CHIKV infection, the virus is sensed by pDCs in an indirect manner that results in selective IRF7 activation and type-I IFN production without the involvement of other inflammatory cytokine responses (89).

NK cells are also a major line of defense against the virus, displaying a marked change in surface receptor repertoire with increased expression of NKp44, CD57, ILT2, CD8α, and NKG2C and decreased expression of NKp30, NKp46, NKG2A, and CD16. Specifically, NKG2C cells undergo rapid expansion during the early acute phase of CHIKD and exert strong cytolytic response upon infected cells (90). As the disease progresses in CHIKD, terminally differentiated NK cells mature into CD56^dim^CD57^+^ phenotype and show a strong cytolytic response and enhanced resistance to cytokine stimulation (90, 91). It has been observed that the number of mature NK cells peak in the early acute phase (i.e., day 3 post-onset of symptoms) and their persistence is correlated with the viral load. Additionally, the persistence of these cells is also associated with chronic CHIKD (90).

In addition to the above mentioned pro-inflammatory mechanisms, the acute phase is also mediated by CD8^+^ T cells responses at the early stages of infection while in the later stages it's the CD4^+^ T cells that predominate the repertoire of the immune cells in humans (92). Murine studies have presented with the evidence that both CD4^+^ and CD8^+^ T cells infiltrate CHIKV-infected tissues during the course of infection (93, 94). These infiltrating T cells are understandably involved in mounting site-specific antiviral immune responses, contrary to which CD4^+^ T cells have recently been shown to contribute toward the pathogenesis during CHIKV infection in mice without altering much of the viral titers along with IFN-γ production (95).

During the acute phase, anti-CHIKV IgM could be detected in sera samples of patients and mice from day 3 onwards after the onset of clinical symptoms (1, 96, 97) whereas the anti-CHIKV IgG with neutralizing activity is produced from day 4 (1, 97). Detection of Anti-CHIKV IgG is mostly rare during the initial days (<4 days) of infection (97). Several studies have also established a correlation between antibodies response with cytokine levels, viral titres, and disease progression during the acute and chronic phases (97, 98). Patients with high virus titres in the acute phase or viremic phase triggered the production of Anti-CHIKV IgG3 antibodies which are strongly involved in protection against chronicity or arthralgia conditions whereas patients with low virus titres associated with chronic arthralgia (99). This suggested that high virus titres in the acute phase seem to induce high titer of Anti-CHIKV antibodies which protect against chronic stages of CHIKD (99). Additionally, CD4^+^ null mice showed lower levels of anti-CHIKV antibodies along with significantly reduced neutralizing activity (100).

CHIKV infected cells are killed by the induction of cytolytic mechanisms along with the several antiviral factors by the immune cells, mainly CD8^+^ T cells (92). Naïve CD8^+^ T cells induce a development environment upon activation leading to effector and memory T-cell proliferation and differentiation (1, 59). Once activated, effector CD8^+^ T cells exhibit functions, such as cytotoxicity and cytokine production, against the virus, resulting in its elimination (101). Furthermore, it is known that CD8^+^ T lymphocytes mediate cytolytic activity against target cells in two major pathways either by the release of cytolytic granules through exocytosis or granule independent pathway, in which they bind to the death receptors of the target cells (102). Studies have reported that the peripheral blood of acute and chronic CHIKD patients had no differences in the percentage of CD8^+^ T cells in relation to healthy individuals. Analysis of early activation marker CD69 for CD8^+^ T cells revealed increased CD8^+^ T cell activation in both acute and chronic diseases (103). The study further observed that CD8^+^ T lymphocytes in patients with acute CHIKD have increased secretion of CD107A along with granzyme and perforin expression in comparison to the control group, suggesting that CD8^+^ T cells may have a role in mediating cytolytic killing of CHIKV infected cells during the acute phase of CHIKD (103). Interestingly, the CD8^+^ T cells failed to present any such increase in the expression of granzyme and perforins in patients with chronic disease in comparison to healthy controls (103). Such observations point to the possibility that these mechanisms slow down or even shut off, owing to the process of T-cell exhaustion during the later stages of the disease.

## Post-Acute Phase

### CHIKV Clearance

Clearance of CHIKV from blood paves the path to recovery in CHIKD patients and is comprised of various immune pathways constituting the post-acute phase of CHIKD. This phase is marked by complete clearance of active CHIKV from the body by neutralizing antibodies in the blood and destruction of infected cells by infiltrating NK cells, CD8^+^ T cells, and neutrophils. Detection of neutralizing antibodies could begin from day 4 to 7 post-onset of symptoms and could last up to 3 weeks post clinical presentation. IgM antibodies could be detected during the acute phase of infection, whereas IgG antibodies are detected in the post-acute phase and may last for months (104–106). Titres of neutralizing antibodies during the acute phase could predict the severity of CHIKD (97). Some patients are completely asymptomatic during the post-acute phase and recover completely. However, a majority of patients show temporary improvement in their clinical state and deterioration occurs after a short “healing” period (61). Also, a recent study using a C57BL/6J mice model has correlated viral clearance and development of IgG antibodies with age. It suggests that CHIKV neutralization and IgG titres were higher in 8-week-old mice during the acute phase, whereas CHIKV clearance was not observed until post-acute phase in 20 weeks old mice (107).

In the context of antibodies, detection of anti-CHIKV IgG is mostly rare during the acute phase of infection while detection occurs in the post-acute phase and persists for months or even years (108). Persistence of CHIKV specific IgM antibodies in the blood and synovial fluid is associated with severe chronic CHIKD (109–111). IgG2c isotype is the major class of antibody produced in the case of CHIKV infected mice model due to IFN-γ produced from CD4^+^ T cells (95). Numerous reports have sought to characterize the neutralizing ability of CHIKV specific antibodies, and it has been reported in mice models as a therapeutic treatment against chikungunya persistence (104, 112–118). In accordance with that, mice mutant Rag1^−/−^ which lack B and T cells, leading to high viral titres in various tissues, concluded that chikungunya infection persists in tissues that are controlled by adaptive immune cells (119). Administration of polyclonal anti-CHIKV virus antisera in CHIKV infected RAG1^−/−^ and B cell-deficient μMT mice resulted in clearance of the virus and could be not detected in blood (25). Similarly, administration of human monoclonal CHIKV-specific antibodies in chikungunya infected rhesus monkeys led to inhibition of viral spread and inflammatory responses in various tissues like joints and muscles (116).

Clearance of circulating pathogens from the system, though, generally involves opsonins like pathogen-specific antibodies and complement component 3 (C3) (120–122). However, a recent study reports that clearance of CHIKV and RRV remains unaffected in the C3^−/−^ and B cell-deficient μMT mice, suggesting the role of an uncharacterized innate immune pathway (123–125). The other mechanism of clearance involves scavenger receptors (SRs), which is a non-opsonic mechanism known for the clearance of endogenous and microbial ligands (126, 127). It was found that Kupffer cells found in the liver carry several SRs, out of which SR-A6 (MARCO) was identified as the key factor responsible for CHIKV clearance (123).

### Disease Resolution

Chikungunya disease resolution is a combination of viral clearance from the host and resolution of inflammation, as all the major symptoms witnessed by the CHIKD patients are due to the robust pro-inflammatory host response. As discussed in the earlier parts of the review, viral clearance in the circulatory system is led by neutralizing antibodies, and virus replicating in the tissues is tackled by infiltrating pro-inflammatory cells like macrophages, CD8^+^ T cells, neutrophils, and NK cells. The other arm of disease resolution, resolution of inflammation, is a complex network of events that strives to attain homeostasis post-inflammation and is regulated by a wide range of mediators (128–133), and inadequate release of these mediators-of-resolution may lead to persistence of inflammation (134). In the case of CHIKV, proteomic profiling of CHIKV infected patients revealed a number of factors that mediate chemotaxis of neutrophils and phagocytes to be downregulated. Azurocidin (AZU1), annexin A1 (ANXA1), CTSG, S-100 calcium-binding proteins S100A7, S100A8, S100A9, and transforming growth factor beta 2 (TGFB2) are significantly downregulated mediators of inflammation found in CHIKV patients with 1–10 days post-onset of symptoms (133). However, a phase-specific analysis of these mediators in CHIKV patients would provide better insight into the mechanism underneath the resolution of inflammation upon CHIKV infection. Also, RNA-seq analysis of feet and lymph nodes of CHIKV infected mice highlighted granzyme A as a major promoter of arthritic inflammation (135).

An important feature that decides resolution of the disease, mainly through regulation of inflammation of joints and attainment of homeostasis, is macrophage polarization and phenotype switching from M1 (pro-inflammatory) to M2 (anti-inflammatory). M1 macrophages restrict the proliferation of surrounding cells and damage infected tissues by releasing pro-inflammatory cytokines, whereas M2 macrophages promote multiplication of contiguous cells and tissue repair (136). This polarization and phenotype switching is regulated majorly by IRF/STAT signaling, tissue microenvironment (hypoxia), oligomerization domain (NOD)-like receptors, cytokine GM-CSF, and NF-κB signaling (136, 137) and has been discussed in detail by Dr. Nan Wang (136). Macrophages exhibiting M2-like activation patterns are found in the CHIKV induced musculoskeletal inflammatory lesions (138) and are known to limit the pro-inflammatory immune response. Arginase 1 (Arg1) is an enzyme associated with M2 macrophages; upon genetic deletion of this enzyme tissue pathology was reduced and enhanced clearance of Ross River virus (RRV) was observed in mice model (138). These results suggest that M2 macrophages play an essential role in the resolution of inflammation, but at the same time M2 macrophages might support the persistence of CHIKV in the system leading to chronic disease. This hypothesis is supported by a study performed on a non-human primate model that suggests long term CHIKV persistence in the macrophages (24).

RNA interference (RNAi) controls the spatial and temporal regulation of the complex gene network involved in the immune response (139). Besides a significant function of silencing of genes related to diseases (140), the viral infection is an example of the complex interplay between viral RNA, endogenous miRNA, and host immune factors (141–144). A recent report suggested that CHIKV establishes infections by regulating miRNA expression profile (145). miR15, miR-16, miR-17, let-7e, miR-125, miR-99, and miR-23a are altered during CHIKV infection which indicates that these act as biomarkers in chikungunya infection (146). Target analysis suggests that targets of these miRNAs are involved in the RIG-1 pathway, TGF-beta-signaling pathway, JAK–STAT-signaling pathway, MAPK-signaling pathway, cytokine–cytokine receptor interactions, and Fc gamma R-mediated phagocytosis (145). In addition, CHIKV infection in human synovial fibroblasts triggers the expression of miR-146a which downregulates the expression of TRAF6, IRAK1, and IRAK2 that in turn leads to decreased activation of NF-κB pathway by a negative feedback loop and promotes CHIKV replication (147, 148). Likewise, miRNAs are also known to participate in macrophage polarization. For example, miR-146a directly binds to the IRF transcription factors and (149, 150) leads to suppression of inflammatory responses by promoting M2 switching. Similarly, miR-210 targets the NF-κB factors and elicits the suppression of M1 switching, resulting in suppression of inflammation (151–153).

Other key players of CHIKD resolution are subsets of T cells, the regulatory T cells (Tregs), and Th17 cells. Differentiation of naïve Th cells into Th17 and Tregs is a common signaling pathway arbitrated by TGF-β; however, the activation of these cells depends on the pro-inflammatory signals which also regulate the fate of these cells in a reciprocal manner (154). In the presence of IL-6 or IL-21, along with TGF-β, naïve Th cells differentiate into Th17 cells, but in the absence of proinflammatory signals, TGF-β drives Treg differentiation (155–157). Th17 cell produces IL-17, IL-22, and IL-23 that attracts neutrophils and hence promotes inflammation at the site of infection (158). In contrast, Treg produces cytokines IL-10 and TGF-β, which suppresses the activity of various immune cells and thereby induces anti-inflammatory responses. Expansion of Tregs, governed by enhanced IL-2 levels and antibody-mediated signaling, also inhibits CD4^+^ effector T cell repertoire, resulting in alleviation of joint pathology (159). In this way, these two cells function in a yin and yang manner and maintain immune homeostasis during infection. It has been shown that the imbalance in cytokines, such as the increase in the concentration of IL-6 along with TGF-β which results in naïve Th cells differentiating into Th17 cells (160), is a key player in arthritis and rheumatism (161). Several studies have shown a significant elevation in the levels of IL-1β, IL-6, and IL-17 associated with Th17 cells in both CHIKV infected humans and CHIKD mice models (92, 93, 162, 163). Conversely, a study conducted on the patient sera samples from Thailand 2009–2010 outbreak showed no significant variations in IL-17 levels as compared to the healthy controls (164). Recent research on flavivirus using mouse model displaying the genetic diversity of the human population has, however, shown that the susceptibility of the host is not only affected by the viral sequence itself but is also controlled by multiple genes of the host (165). These studies suggest the need for detailed research unveiling the fundamental mechanisms involving immune cells and host genetic diversity as a whole in CHIKD resolution and inflammation.

Another class of regulatory cells that modulates the immune response by producing anti-inflammatory cytokines such as interleukin-10 (IL-10), IL-35, and transforming growth factor β (TGF-β) is a subset of B cells called regulatory B cells (Bregs) (166, 167). Bregs cells inhibit the expansion of pro-inflammatory lymphocytes and other pathogenic T cells (166) by exerting the inhibitory effect on production of pro-inflammatory molecules, such as TNF-α, IFN-γ, and IL-17 from CD4^+^ T cells (167). However, Bregs also regulate the immune response by modulating the induction of cell death and IgM production, as reported both in humans and mice models (168–171). CD9^+^ Bregs stimulate differentiation of CD4^+^ T cells into Tregs by producing high levels of TGF-β (171) which further alleviates the production of TNF-α and induces apoptosis in pro-inflammatory CD4^+^ T cells. Production of IgM persuades elimination of apoptotic bodies, leading to reduction of pro-inflammatory mediators (172, 173). Bregs have been studied for their role in rheumatoid arthritis (RA) and its regulation seems to be one of the key players that enhance RA disease (174, 175). Considering that chikungunya-induced arthritis is an inflammatory response triggered by the virus that persists in joint tissues resulting in chronic rheumatoid disease (1), it is possible that Bregs might be involved in the regulation of chikungunya-induced RA via modulating inflammatory responses.

## Chronic Phase

In several CHIKD cases, inflammation causing joints pain might persist for several months or years, resulting in symptoms similar to rheumatoid arthritis, and is termed as the chronic phase of CHIKD (176–178). Typically, the chronic phase is characterized by joint swelling, joint stiffness, arthralgia, and tendonitis/tenosynovitis (119). Serologically, it marks a significant reduction of the virus as well as of viral RNA, reduced osteoprotegerin, and increased IL-6 and RANKL (receptor activator of NF-κB) ferritin, CRP (C-reactive protein), CXCL9, CXCL1, CHIKV-specific IgG titer IL-12 GM-CSF, IL-17, IL-27, IL-29, IL-8, MCP-1, MIP, CD95/CD95L, CXCL-9, and CXCL-10 (26, 92, 103, 162, 179–182). At this stage, CHIKV virus particles/viral RNA is cleared from the blood; however, studies have reported the persistence of CHIKV RNA in the macrophages (24) and fibroblast during chronic phase (183). Further, the viral dsRNA (intermediate stage during replication) found in the monocytes/macrophages of joints has been reported to trigger an arthritogenic response by activating NF-κB, both in *in vitro* studies (76), as well as in ankles and wrists of mice Rag^−/−^ (119). Apart from RNA intermediates, studies have reported the persistence of CHIKV specific proteins in host cells over an extended period of time. CHIKV-nsP3 was found to be present as granules along the cell membrane in cells in persistent infection (33), while in another study, CHIKV-capsid proteins were evident in CHIKV infected mice as late as 60 days post-infection, suggesting the active translation of viral proteins during the chronic phase of CHIKD (25).

CD4^+^ and CD8^+^ T cells infiltrate the joints in CHIKD, causing inflammation. Specifically, CD4^+^ T cells are majorly responsible for joint swelling and inflammation and possibly mediate inflammatory pathways through CD4^+^ T-cell lineages, T_H_1 and T_H_2 cells that secrete anti-osteoclastogenic cytokines, IFN-γ, and IL-4. The infiltration of T_H_ 17 cells into the inflammatory joint links the abnormal T-cell response to bone damage in arthritis (184, 185). One study found increased expression of CD95/CD95L in CD4^+^ cells (92). In CD8^+^ cells, although cells are nearly constant from healthy to acute to chronic phase, the persistent exposure of antigens to CD8^+^ T cells leads to altered expression of biomarkers like CD69A, CD107A, and IL-17A (103). As CD8^+^ T cells only express CD95L after activation (186), it can be safely assumed that these cells stay activated through the chronic phase, hence having a role in disease resolution and chronicity. Furthermore, effective immune cell activation drives the initial antiviral response leading to T-cell exhaustion, and this may be linked to viral persistence observed in the chronic phase (26). Additionally, the absence of CD8^+^ T cells in synovial tissue may also be a contributing factor toward the persistence of CHIKV (26).

**Figure 3:**
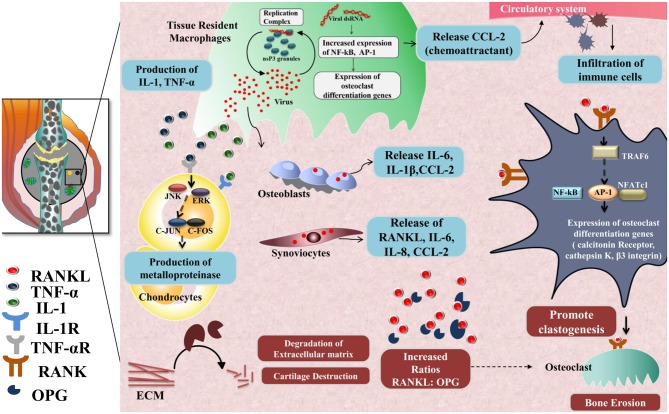
Chronic phase-CHIKV mediated joint pathology: in the joints, osteoblast cells (Bone-forming cells) are susceptible to CHIKV infection causing over-expression of IL-6 and RANKL and inhibition of osteoprotegerin (OPG). Also, fibroblast-like synoviocytes (HFLS) that are present in the synovial membrane lining the synovial joints, secrete enhanced levels of RANKL, IL-6, IL-8, and (MCP-1), upon CHIKV infection resulting in higher RANKL: OPG ratio, which favors osteoclast formation from precursor cells. RANKL and OPG maintain bone homeostasis as RANKL promotes osteoclastogenesis. RANKL recruits TRAF6 which induces auto amplification of NFATc1 (a transcription factor) via NF-κB and c-fos pathway and dependent on calcium signaling of ITAMs clastogenesis. NFATc1 in complex with AP-1 regulates the expression of genes involved in osteoclast differentiation e.g., calcitonin receptor, cathepsin K, and β3 integrin. Secretion of IL-1 and TNF-α by CHIKV infected resident macrophages phosphorylate JNK and ERK which in turn activates AP-1 family member, c-jun, which dimerize with c-fos and induce transcription of matrix metalloproteinases (MMP) that degrades components of extracellular matrix by collagenase activity.

Another hallmark of the chronic phase is bone erosion and degradation of extracellular matrix in joints, causing chronic arthralgia in CHIKD patients. Key players of the mechanism involved in bone erosion are resident macrophages, CCL2, IL-6, RANKL (187), osteoprotegerin, TRAF6, NFATc1, calcitonin receptors, cathepsin K, and β3 integrin (184, 188); degradation of the extracellular matrix, meanwhile, is mediated by several factors such as fibroblast-like synoviocytes IL-1, TNF-α, Janus kinase, ERK, c-jun, and c-fos. These molecules, in turn, activate matrix metalloproteinases (MMP) in chondrocytes that promote extracellular matrix degradation during the chronic phase (189) (Figure 3).

## Conclusion

Complexities in CHIKD immunopathology in the host begins from the point of mosquito saliva that may contain both the virus as well as the viral antibodies that may affect the virus pathogenicity when delivered into the host during a bite. Furthermore, the immunomodulatory role of mosquito saliva is strikingly an understudied phenomenon in CHIKV infection. Does the mosquito saliva possess factors that can actively modulate viral pathogenesis? Can these molecules be utilized for developing novel antiviral strategies? How exactly does the presence of viral antibodies from previous bites affect CHIKV pathogenicity during the next bite to a naïve individual? Even though some of these aspects have been looked into, in-depth studies are necessary to address these concerns more effectively. Additionally, answering some of these questions may help in arriving on novel transmission blocking strategies.

Once the infection is established, the innate and adaptive host immunity plays definitive roles in the progression of the disease that we know to be both protective as well as pathogenic in its response. These responses decide the clinical presentation of the disease at the febrile acute phase in terms of peak viremia, small joint and large joint involvement, as well as disease resolution during the post-acute phase. The current understanding of the subject suggests that arthritogenic symptoms in the acute phase of CHIKD are primarily due to pro-inflammatory host responses that could lead to the possible association of severe chronic cases with autoimmunity. Can we derive more knowledge regarding this association by looking into other similar autoimmune disorders such as rheumatoid arthritis? Will employing animal models specific to autoimmune disorders help in answering some immunological questions pertaining to immune response of CHIKV-induced arthritis? Can these systems recapitulate the actual pathogenesis seen in humans? How well are we using the clinical evidence to understand disease progression in the case of CHIKD? How do we use the information pertaining to inflammation more usefully in understanding CHIKD immunopathology? Can we devise intervention approaches based on this information pertaining to inflammation specifically to alleviate the arthritic phase?

The resolution of CHIKD is not yet fully understood and would be the most interesting area to be studied in future, as there are several well-established mediators of inflammation that have not been studied in the context of CHIKV chronic infections. This review has strived to emphasize the various immunological aspects that may lead to disease resolution/progression in CHIKD and highlight the lacuna present currently in the field.

## Author Contributions

SS: conceptualization, supervision, and funding acquisition. PS, AK, AH, DM, RK, CS, and SS: methodology, data curation, writing-original draft preparation, and writing-review and editing.

## Conflict of Interest

The authors declare that the research was conducted in the absence of any commercial or financial relationships that could be construed as a potential conflict of interest.
